# Cardiac Tamponade: A Late and Rare Finding of Hypothyroidism

**DOI:** 10.7759/cureus.70617

**Published:** 2024-10-01

**Authors:** Taysir Al Janabi, Vishal Gupta, FNU Naintara, Jacob Seidenberg, Abhinav Hoskote

**Affiliations:** 1 Internal Medicine, WellSpan York Hospital, York, USA

**Keywords:** cardiac tamponade, hashimoto thyroiditis, hypothyroidism, levothyroxine, liothyronine, pericardial effusion

## Abstract

“Myxedema heart” is a rare syndrome that results from severe untreated hypothyroidism. It is characterized by relative bradycardia, low-voltage electrocardiogram (EKG), cardiomyopathy, and pericardial effusion. Here, we present a case of cardiac tamponade in untreated hypothyroidism. A 49-year-old male with no prior medical or psychiatric history presented to the Emergency Department for cognitive slowing, weakness, and inappropriate behavior for several weeks. Vital signs were significant for bradycardia with normal blood pressure. Examination revealed that the patient was disheveled and alert but forgetful with slow speech, along with ataxia and muscle wasting.

The EKG showed low-voltage sinus bradycardia. Laboratory workup was notable for an elevated thyroid-stimulating hormone (TSH) level of more than 100 mcIU/mL, with low free T4 and T3. Anti-thyroid peroxidase (TPO) and thyroglobulin antibodies were elevated, confirming a diagnosis of Hashimoto’s thyroiditis.

Treatment with intravenous levothyroxine, triiodothyronine, and hydrocortisone was started. An echocardiogram revealed a large circumferential pericardial effusion with evidence of tamponade. A pericardial drain was placed, and it drained 980 mL of sanguineous fluid. Serial echocardiograms showed a stable posterior effusion with no recurrence of the anterior pericardial effusion. Gradually, the patient’s mentation, hemodynamics, and electrolyte levels improved. He was discharged on oral levothyroxine. TSH values showed sequential improvement.

A large pericardial effusion with tamponade is a rare, life-threatening complication of untreated hypothyroidism. Clinicians must promptly identify myxedema and replace thyroid hormone to prevent the progression of pericardial effusion to tamponade and reverse its pathophysiology, maintaining a high level of suspicion in those at risk of poor healthcare access or inadequate health literacy.

## Introduction

Thyroid hormones are essential in the development and function of the cardiovascular system. The most common cause of hypothyroidism in the United States is Hashimoto thyroiditis, an autoimmune disorder. The classic cardiovascular signs of hypothyroidism are diastolic hypertension, sinus bradycardia, dyslipidemia, myxomatous valvular changes, and pericardial effusion [1]. The incidence of pericardial effusion in overt hypothyroidism is 3-6%. However, cardiac tamponade in newly diagnosed hypothyroidism is rare and occurs mainly in severe hypothyroidism [2]. The pathophysiology behind pericardial effusion and tamponade is attributed to an increase in albumin permeability in the pericardial capillaries - a process that is induced by the impact of low thyroid state indirectly on the histamine release or directly on the pericardial capillary endothelium - along with the impairment in lymphatic drainage [3]. Here, we present a rare case of cardiac tamponade in untreated primary hypothyroidism.

## Case presentation

A 49-year-old male with unremarkable past medical history was brought to the Emergency Department (ED) due to several weeks of cognitive slowing, weakness, and inappropriate behavior, involving defecating on himself, eating old food, and barricading in his room. Vital signs were significant for bradycardia of 40-50 beats per minute (bpm), with normal temperature and blood pressure. On examination, the patient was noted to be disheveled and alert but forgetful with slow speech. Additionally, ataxia and muscle wasting were present.

**Figure 1:**
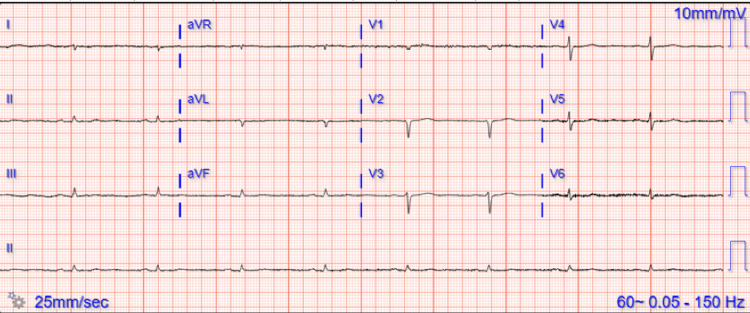
EKG upon presentation showing sinus bradycardia with low-voltage QRS. EKG, electrocardiogram

Electrocardiogram (EKG) showed sinus bradycardia with low voltage (Figure 1). Laboratory workup was notable for hemoglobin of 8.8 g/dL, mild hyponatremia of 133 mmol/L, and blood glucose level of 75 mg/dL; additionally, thyroid-stimulating hormone (TSH) was incidentally >100 mcIU/mL, free thyroxine (T4) was low at <0.3 ng/dL, and free triiodothyronine (T3) was low at 1.60 pg/mL (Table 1). An intravenous levothyroxine injection of 200 mcg was given.

**Table 1:** Laboratory findings and their reference values TSH, thyroid-stimulating hormone; TPO, thyroid peroxidase

	Laboratory findings	Reference range
Hemoglobin (g/dL)	8.8	13.0–17.3
Sodium (mmol/L)	133	135–145
Blood glucose (mg/dL)	75	70–139
TSH (mcIU/mL)	>100	0.30–5.00
Free thyroxine, T4 (ng/dL)	<0.3	0.6–1.6
Free triiodothyronine, T3 (ng/dL)	1.60	2.50–3.90
Thyroglobulin (ng/mL)	<0.1	1.6–50
TPO antibody (IU/mL)	574	≤9
Thyroglobulin antibody (IU/mL)	>2,500	≤4

**Figure 2:**
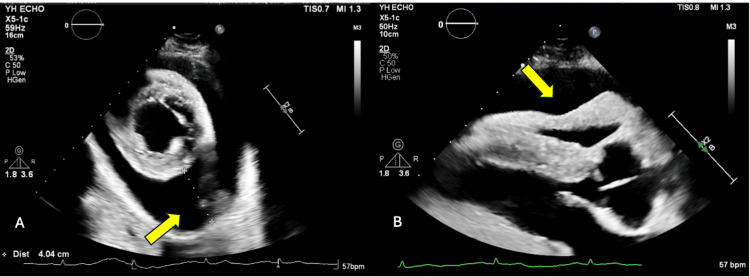
Echocardiogram views: (A) parasternal short axis (PSAX) showing a very large pericardial effusion circumferentially and (B) parasternal long axis (PLAX) showing right ventricular diastolic collapse.

The next day, the patient became more lethargic, bradycardic, and hypoglycemic. He was admitted to the intensive care unit (ICU) with concern for myxedema coma. Thyroglobulin was suppressed at <0.1 ng/mL. TPO antibody as well as thyroglobulin antibody were significantly elevated at 574 IU/mL and >2,500 IU/mL, respectively, values consistent with Hashimoto's thyroiditis. A thyroid ultrasound showed a normal-sized thyroid gland with heterogeneous echogenicity. A transthoracic echocardiogram revealed a very large pericardial effusion circumferentially with evidence of cardiac tamponade (Figure 2), including right ventricular compression, a collapse of the right atrium exceeding one-third of the cardiac cycle, and respiratory variation of greater than 25% variability noted on the mitral inflow Doppler.

**Figure 3:**
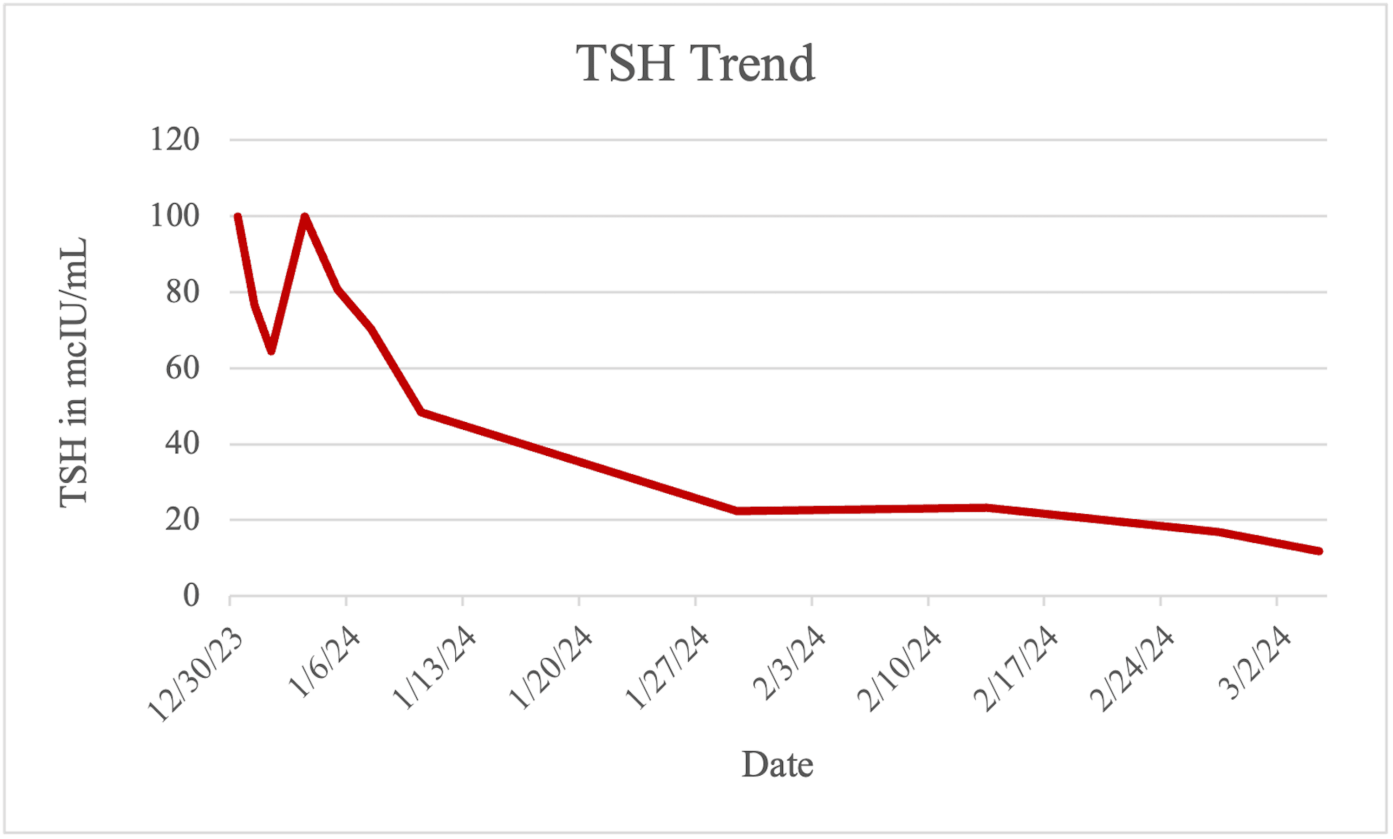
Sequential improvement in TSH values TSH, thyroid-stimulating hormone

The patient was started on liothyronine 5 mcg three times daily, intravenous (IV) levothyroxine 100 mcg daily, and stress dose of hydrocortisone 100 mg IV three times daily. The patient had a pericardial drain, draining 980 mL of sanguineous fluid. After clamping trials, the drain was removed 48 hours later. Serial echocardiograms showed a stable posterior effusion with no recurrence of the anterior effusion. The patient’s mentation, hemodynamics, and electrolyte derangements improved gradually, and he was discharged on oral levothyroxine 125 mcg daily. TSH values showed sequential improvement (Figure 3).

## Discussion

Hypothyroidism is one of the most commonly encountered clinical conditions, with a prevalence of 4% to 10% in the general population [4]. Its etiology is also geographically variable. For example, the most common cause of hypothyroidism in limited-resource countries is iodine deficiency, while in rich-resource countries, such as the United States, autoimmune disease is the leading cause, such as Hashimoto thyroiditis [5]. Additionally, the broad non-specific symptoms of hypothyroidism have led to reliance on biochemical testing to make a diagnosis [6]. Fatigue, constipation, and cold intolerance are the most common symptoms and signs. Cardiovascular effects such as bradycardia, hypertension, dyslipidemia, pericardial effusions, and cardiac tamponade have been reported in severe hypothyroidism.

Classically, hypothyroid individuals who develop pericardial effusion or cardiac tamponade present mostly with shortness of breath, cough, and chest pain, which were reported to be around 61.1%, 25%, and 13.9%, respectively [7,8]. Our patient's presentation was quite different as he presented with cognitive impairment and inappropriate behavior; further investigation revealed bradycardia in the setting of hypothyroidism, and his pericardial effusion did not exhibit the classic symptoms of effusion. His pericardial effusion was an incidental finding.

Literature shows a temporal association between pericardial effusion and severity of disease, as suggested by elevated TSH, which is found more frequently in congenital and long-standing cases of untreated hypothyroidism [9,10]. The majority of hypothyroid individuals with pericardial effusion have a TSH value of less than 30 mcIU/mL. However, our case presented with a TSH value of more than 100 mcIU/mL [3]. The large amount of effusion and severely elevated TSH suggest that the pericardial effusion had progressed very slowly over a long period, allowing the pericardial sac to stretch and accommodate almost a liter of fluid.

The current literature reports that 16% of the cases of pericardial effusions result in serosanguineous fluid accumulation, with the most common cause being trauma to either the heart or surrounding structures [11]. The research team believes that the reason behind the serosanguineous pericardial fluid is due to pericardiocentesis, as the pathology report was unremarkable.

Levothyroxine is the usual treatment for hypothyroidism, which reverses the progression of pericardial effusion and prevents cardiac tamponade; it is estimated that complete resolution can occur within 4 to 26 weeks without any invasive procedure [12]. However, that was not the clinical course in our patient, as he showed evidence of tamponade and underwent pericardiocentesis with subsequent placement of a pericardial drain. Additionally, our patient received liothyronine in addition to the standard treatment of levothyroxine as liothyronine has rapid action and more impact compared to levothyroxine; the response was manifested biochemically by reducing TSH by more than 50% in two weeks. While professional societies are against the use of combined levothyroxine and liothyronine because of a lack of long-term safety data and the effectiveness of the combination, they still recommend that further research on combined thyroid replacement therapy should focus on improved clinical outcomes and patient satisfaction with the treatment [13].

## Conclusions

Hypothyroidism can affect virtually any organ, and the pericardium is no exception. While small pericardial effusions are common with chronic hypothyroidism, a large pericardial effusion with tamponade is a rare, life-threatening complication that requires prompt recognition and management. Clinicians must promptly identify “myxedema heart” and replace the thyroid hormone to prevent the progression of pericardial effusion to tamponade, especially maintaining high suspicion in those at risk of poor access to healthcare or inadequate health literacy.
